# Salvage thoracic surgery in patients with lung cancer: potential indications and benefits

**DOI:** 10.1186/s13019-018-0693-x

**Published:** 2018-01-22

**Authors:** Erkan Kaba, Mehmet Oguzhan Ozyurtkan, Kemal Ayalp, Tugba Cosgun, Mazen Rasmi Alomari, Alper Toker

**Affiliations:** 1 Department of Thoracic Surgery, Istanbul Bilim University Medical Faculty, 34381 Sisli, Istanbul, Turkey; 2 Department of Thoracic Surgery, Group Florence Nightingale Hospitals, Istanbul, Turkey

**Keywords:** Non-small-cell lung cancer, Salvage surgery, Pulmonary resection, Survival

## Abstract

**Background:**

To investigate the feasibility and efficacy of salvage lung resection and describe the possible indications and contraindications in patients with primary lung cancer.

**Methods:**

Thirty patients undergoing anatomical salvage lung resection were classified into three groups: GI, patients with progressive lung tumor despite definitive chemo- and/or radiotherapy; GII, patients who underwent emergency resection; and GIII, patients in whom neoadjuvant or definitive chemo- and/or radiotherapy was contraindicated because of severe comorbidities. The groups were compared based on, peri- and postoperative factors, and survival rates.

**Results:**

The morbidity rate was 70%. Revision surgery was required in 23% of patients. Morbidity was affected by lower hematocrit and hemoglobin levels (*P* = 0.05). Mean hospital stay was 11 ± 4 days, which was longer in patients in whom complications developed (*P* = 0.0003). The in-hospital or 30-day mortality rate was 3%. Mean relapse-free survival and overall survivals were 14 ± 12 and 19 ± 13 months.

**Conclusion:**

Patients with progression of the persistent primary tumor after definitive chemo- and/or radiotherapy can undergo salvage lung resection with acceptable mortality and high morbidity rates, if the tumor is considered resectable. Other indications may be considered for salvage lung resection based on each patient’s specific evaluation.

## Background

Lung cancer is a leading cause of death worldwide. Although surgery is the standard of care for patients with operable early-stage lung cancer, definitive chemoradiation has been introduced for resectable tumors in high-risk patients or for unresectable locally advanced tumors [1]. Local relapses have been reported to develop within 2 years after definitive chemoradiotherapy in >30% of patients [2]. In such a situation, treatment options are repeat irradiation, chemotherapy, cryo- and radiofrequency ablation, and observation only [3].

Salvage operations are occasionally performed in selected patients with advanced colon and esophageal cancer and malignant mediastinal tumors, such as thymoma [4]. Recently, this term has been adopted for lung cancer. Some retrospective studies demonstrated that salvage lung resection (SLR) can improve survival with acceptable surgical adverse events [5–7]. However, the experiences are limited. The impact of SLR on the early and long-term effects and the indications for SLR remain unclear.

In this single-institution study, we reported patients undergoing SLR and investigated the feasibility and efficacy of the procedure. We also described the possible indications and contraindications for SLR.

## Methods

This study was approved by the institutional review board of Istanbul Bilim University, and the need for individual patient consent was waived. Among 496 patients who underwent anatomical pulmonary resections because of primary lung cancer at our institution between January 2011 and December 2016, we retrospectively evaluated 30 (6%) who underwent SLR. SLR was defined as anatomical resection in patients who had progression of the persistent primary lung tumor after definitive chemo- and/or radiotherapy (GI, 22 patients); in those who underwent resection due to an emergency situation that could not be treated with other major therapeutic modalities, such as hemoptysis, bronchoesophageal or bronchopleural fistula, and severe infectious situations, including lung abscess or empyema (GII, three patients); and in those who should have undergone neoadjuvant or definitive chemo- and/or radiotherapy, but these were contraindicated because of severe comorbidities despite the clinical diagnosis of stage IIIA, IIIB, or IV disease, as previously described (GIII, five patients) [4, 8].

Patients had been discussed during our weekly multidisciplinary tumor board, in which thoracic surgeons, chest physicians, pulmonary oncologists, radiation oncologists, radiologists, and nuclear medicine specialists took part. Patients were chosen as candidates for SLR when their physical status and cardiopulmonary functions, as determined using lung function tests, exercise tests, and cardiac evaluation, were sufficient for them to undergo resection, and when complete resection of all suspicious or proven disease was technically feasible. However, patients in GIII were accepted to have borderline pulmonary or cardiac levels at the time of evaluation. For patients in GI, the operation was performed at least 6 months after definitive chemo- and/or radiotherapy. SLR entailed appropriate anatomical lung resection and mediastinal lymph node dissection. All patients in GI and selected patients in the other groups underwent reinforcement of the bronchial stump using any of the following: intercostal muscle flap, diaphragm, pleura, and thymus. In the postoperative period, all patients received similar drug regiments including bronchodilators, analgesics, expectorants and antibiotics (ampicillin-sulbactam and ciprofloxacin).

Data retrieved from the database included age, sex, comorbidity, postoperative pathology, N- and T-stages of the tumor, indication for operation, type of operation, preoperative levels of hemoglobin, hematocrit (%) and albumin, respiratory function tests [forced expiratory volume in one second (FEV1) and diffusion capacity of the lung for carbon monoxide (DLCO) values], pulmonary arterial pressure and ejection fraction values, length of hospital stay, mortality, morbidity, and follow-up records. Surgical complications were classified based on the proposal made by Dindo et al. [9]

The date of surgery was used for follow-up measures. The patients underwent a chest and abdomen physical examination. The initial computed tomography (CT) scan was performed 3 months postoperatively, and chest and upper abdomen CTs were performed every 6 months for the first 2 years and once yearly thereafter for 5 years. Overall survival was calculated from the time of surgery. Relapse-free survival rate was calculated from the date of surgery to the date of recurrence.

The three groups were compared based on the aforementioned parameters. The data were collected and analyzed using Excel software (Microsoft Corp., Seattle, WA, USA). Descriptive statistics were used to report the means and standard deviations of the continuous variables and the number and percent of categorical variables. Categorical data were compared using the Fisher’s exact test and *t*-tests, as appropriate. To test significant differences between the groups, one-way analysis of variance followed by Tukey’s test was used for normally distributed variables and the Kruskal–Wallis test was used for non-normally distributed variables. *P* ≤ 0.05 was considered statistically significant.

## Results

Table 1 demonstrates the characteristics of the patients. Mean patient age at resection was 63 ± 7 years. Most were male patients (*n* = 26, 87%). A total of 18 patients (60%) had comorbidities, including chronic obstructive pulmonary disease in eight, coronary artery disease in seven, presence of cancer other than lung cancer in three, severe hyperthyroidism in one, and severe liver insufficiency in one. There were no significant differences between the groups in terms of age, sex, and presence of comorbidities.

**Table 1:** Characteristics of the patients*

	Total (*n* = 30)	Group 1 (*n* = 22)	Group 2 (*n* = 3)	Group 3 (*n* = 5)
Age (years, ± SD)	63 ± 7	63 ± 8	64 ± 3	62 ± 8
Male/Female	26/4	21/1	3/0	2/3
Presence of comorbidity (*n*, %)	18 (60%)	11 (50%)	2 (67%)	5 (100%)
Operation type (*n*)				
Lobectomy	14	11	0	3
Greater resection	11^a^	8^b^	2^c^	1^d^
Segmentectomy	5	3	1	1
Extended resection (*n*, %)**	16 (53%)	13 (59%)	1 (33%)	2 (40%)
Length of hospital stay (mean days ± SD)	11 ± 4	10 ± 4	14 ± 2	13 ± 9
Mortality (*n*, %)	1 (3%)	0	0	1 (20%)
Morbidity (*n*, %)	21 (70%)	14 (64%)	3 (100%)	4 (80%)
Overall survival (mean months ± SD)	19 ± 13	22 ± 13	13 ± 5	6 ± 2
Relapse-free survival (mean months ± SD)	14 ± 12	16 ± 13	9 ± 4	6 ± 2

^a^Pneumonectomy, 8; bilobectomy, 2; lobectomy + segmentectomy, 1

^b^Pneumonectomy, 6; bilobectomy, 1 lobectomy +segmentectomy, 1

^c^Pneumonectomy, 1; bilobectomy, 1

^d^Pneumonectomy, 1

SD: Standart Deviation

*P > 0.05 for all parameters

**Including sleeve resection (bronchial or arterial), chest wall resection, pericardial resection and reconstruction, right atrial resection, and diaphragmatic resection and reconstruction

A total of 22 patients (73%) underwent SLR because of either progression of the primary lung tumors after previous chemotherapy (7 patients) and/or definitive radiotherapy with concurrent chemotherapy (10 patients) or recurrent primary lung tumor after previous pulmonary resections and adjuvant treatment (5 patients). Five patients (17%) underwent SLR, because they were considered unsuitable to undergo chemotherapy or radiotherapy because of severe comorbidities. SLR was performed as palliative intent in three patients (10%; two had septic pulmonary abscess and one had severe empyema).

Of the 30 operations, three were performed in 2013, seven in 2014, five in 2015, and 15 in 2016, and they included 14 lobar resections, 11 greater resections (eight pneumonectomies, two bilobectomies, and one lobectomy combined with segmentectomy), and five segmentectomies. A total of 16 patients (53%) required extended surgery (bronchial and/or arterial sleeve in six, chest wall resection in four, pericardial resection and reconstruction in three, right atrial resection in two, and diaphragmatic resection and reconstruction in one). Althought most patients in GI required extended resections compared with the other groups, the difference was insignificant.

Overall, 18 patients (60%) had squamous cell carcinoma, 11 (37%) had adenocarcinoma, and one (3%) had adenosquamous cell carcinoma. R0 resections of bronchial and/or vascular margins were performed in 28 patients (93%). Only two patients (7%) had incomplete resections, and they both had positive arterial vascular margins. Final pathological examination demonstrated viable tumor cells in 27 patients (90%). T-stage was T1 in four patients, T2 in six, T3 in 12, and T4 in five. N-stage was N0 in 16 patients, N1 in five, and N2 in six.

Morbidity occurred in 21 patients (70%). According to the Clavien–Dindo classification, the rates of grades 1, 2, and 3 complications were 6.7%, 36.6%, and 26.7%, respectively. Pneumonia was the most common complication (40%), followed by arrhythmia (20%). Among seven patients (23.3%) who underwent revision surgery, five had intrathoracic hematoma and underwent exploration via thoracotomy, which did not reveal major vascular or active bleeding. One patient who had undergone an emergency right upper bilobectomy because of septic pulmonary abscess required revision surgery because of a bronchopleural fistula. One patient in GI who underwent right lower bilobectomy with pericardial resection and reconstruction suffered pericardial graft infection requiring reoperation. Morbidity was not related to age, indication for surgery, type of surgery, presence of comorbidities, requirement of extended resections, results of respiratory function tests, or echocardiographic parameters. Patients who suffered complications had significantly lower hematocrit (34% vs. 37%, *P* = 0.04) and hemoglobin (11 vs. 12 g/dL, *P* = 0.05) levels.

There were no intraoperative deaths. One patient (3%) died of pneumonia 28 days postoperatively. This patient had lower respiratory functions [DLCO, 34%; FEV1, 41%; and maximum volume of oxygen (VO2 max), 13.2 mL/kg/min]. She was considered unsuitable to undergo chemotherapy. She also had a history of lymphoma and treatment, including radiotherapy, which compromised pulmonary function. She underwent double-sleeve left upper lobectomy, and she was one of the aforementioned patients who required revision surgery because of intrathoracic hematoma.

Mean hospital stay was 11 ± 4 days, which was significantly longer in patients in whom a complication developed (12 vs. 7 days, *P* = 0.0003). The hospital stay was not significantly longer in patients with grade 3 compared with those with grade 1 or 2 complications according to the Clavien–Dindo classification (16 vs. 12 days, *P* = 0.06).

Median postoperative follow-up for the surviving 29 patients was 15 (range, 2–50) months. A total of 15 patients (50%) were alive without disease at the end of the study. In 12 patients (40%), locoregional recurrence and/or distant metastasis developed at a median of 10 (range, 6–19) months. Among these patients, five had local recurrence in the ipsilateral thoracic cavity within a median of 6 (range, 6–18) months, whereas seven had metastasis to the brain (three), bones (two), and multiorgan systems (two) at a median of 11 (range, 6–19) months. These patients received further chemotherapy and/or radiotherapy when appropriate, and nine of them died at a median of 24 (range, 8–30) months. Two patients (7%) without evidence of recurrence or distant metastasis died of cardiac failure 2 and 3 months postoperatively. These patients had undergone right upper lobectomy with chest wall resection and reconstruction and partial vertebral corpus resection, and left upper lobe bronchial sleeve lobectomy, respectively. Mean relapse-free and overall survivals were 14 ± 12 months and 19 ± 13 months. Survival rates were not affected by factors analyzed in this study.

Among two patients with positive arterial resection margins (both had negative bronchial margins), one had a recurrence at 6 months, and died of metastasis at 26 months postoperatively, whereas the other had a recurrence at 18 months, and died of metastasis at 30 months. Although patients in GI had longer overall and relapse-free survivals compared with the other groups, the results were insignificant (22 vs. 13 and 6 months; 16 vs. 9 and 6 months, respectively, *P* > 0.05).

## Discussion

We evaluated the feasibility and efficacy of SLR in patients with primary lung cancer and compared our results to those of previous reports. SLR in the field of lung cancer treatment is not yet a commonly accepted treatment modality, and the definition of SLR varies among several investigators. The term SLR is generally used when the operation is performed after stereotactic radiotherapy [10, 11] or chemotherapy [12, 13]. A review of the literature mostly reveals cohorts of patients undergoing SLR after local recurrence or persistent tumor following chemoradiotherapy [3, 14–16], radiotherapy, or steretotactic radiotherapy [7, 17, 18]. Table 2 demonstrates several studies published since 2014, including the current study.

**Table 2:** Recent studies concerning SLR

Authors	Years	*N*	Indication	Timing of surgery in weeks (range)	Mortality (%)	Morbidity (%)	Follow-up (months)	Overall survival (months)	Relapse-free survival (months)
Uramoto et al. [12]	2014	8	Mixed	n.g	0	25	14	n.g.	5.9
Yang et al. [7]	2015	31	Recurrent or persistent tumor after radiotherapy	18 (8–111)	0l	48	n.g.	32	10
Dickhoff et al. [14]	2016	15	Local recurrence and persistent tumor after chemo-, radiotherapy	21 (3–95)	6.7	40	12.1	46	43.6
Schreiner et al. [3]	2016	9	Local recurrence after chemo-, radiotherapy	30 (12–165)	11	22	30	23	21
Verstegen et al. [17]	2016	9	Recurrent or persistent tumor after stereotactic radiotherapy	n.g.	0	33	19	26	n.g.
Shimada et al. [15]	2016	18	Local recurrence and persistent tumor after chemo-, radiotherapy	38 (3–282)	0	28	47	n.g.	n.g.
Mizobuchi et al. [18]	2016	12	Recurrent or persistent tumor after radiotherapy	96 (36–312)	0	n.s.	18	n.g.	n.g.
Sawada et al. [16]	2017	8	Local recurrence and persistent tumor after chemo-, radiotherapy	n.g.	0	38	48	n.g.	n.g
This study	2017	30	Mixed	n.g.	3	70	15	15	11
Only GI patients		22	Recurrent or persistent tumor after definitive chemo-, radiotherapy, or previous surgery and ajduvant treatment	24	0	64	22	22	16

n.g.: not given

The clinical significance of SLR remains controversial, and comparing the results of the aforementioned studies to each other may not be reliable. This is because of the differences in the definition of SLR among investigators and in the patient selection [4]. Therefore, it was proposed that performing SLR might be feasible with appropriate patient selection because the median survival after other treatment modalities generally is <1 year for patients with recurrent local lung cancer [19].

Most of our patients (*n* = 22) underwent SLR because of progresion of the persistent primary lung tumor after previous chemo and/or definitive radiotherapy and because of local recurrence of primary lung tumor after previous surgery and adjuvant treatment. Our study also included patients in whom chemo- or radiotherapy was considered to be contraindicated (*n* = 5) and those requiring palliative resection because of complications of the tumor or treatment (*n* = 3). Similar patient selection criteria were applied in the study reported by Uramoto et al. [12]

There is no exact timing for performing SLR after chemo- and/or radiotherapy. Patients underwent SLR at an interval of 18–96 weeks after completion of the previous treatment (Table 2). Bauman et al. [5] recommended not delaying the operation further because the increased time between completion of radiotherapy and surgery theoretically may cause the development of more fibrosis. Operating on a fibrotic lung, which has brittle and devascularized tissue and obliterated planes, may result in increased risks of fistulae and impaired wound healing. Increasing the duration may cause more difficult identification, manipulation, and dissection of tissues, leading to increased blood loss compared towith standard procedures [20]. However, further delaying SLR after definitive radiotherapy was reported to result in comparable outcomes in terms of complication rates [7]. Shimada et al. [15] reported a large volume of intraoperative blood loss (399 mL) and considerably longer operative duration (5 h).

In our study, SLR was performed at least 24 weeks following the previous definitive chemo- and/or radiotherapy in all patients in GI. There was no mortality in this subgroup, whereas the complication rate was higher (64%) than that in previous reports (Table 2). Another important retrospective study by Bauman et al. [5] demonstrated that patients undergoing SLR had a mortality rate of 4% and a high complication rate of 58%. Concerning the whole study population, our mortality and morbidity rates were 3% and 70%, respectively. We demonstrated that the lower hematocrit and hemoglobin levels were related to the development of complications. No other factors were related to morbidity. The complication rate was insignificantly higher in patients who underwent extended resections (44% vs. 57%). As seen in Table 1, all patients who underwent an emergency operation and 80% of the patients in GIII suffered morbidities compared with GI (64%), but the result was insignificant.

To minimize perioperative complications, Yang et al. [7] proposed that pneumonectomy should be avoided, if possible, and the bronchial stump should be covered using appropriate tissues. Dickhoff et al. [14] reported that the survival rate in patients undergoing lobectomy was higher. They also favored coverage of the bronchial stump, especially in patients undergoing pneumonectomy, none of whom had a postoperative bronchopleural fistula in their series. In our series, we routinely reinforced the bronchial stump. We also performed pneumonectomy in only eight patients, five of whom suffered a complication (three had pneumonia and two had atrial fibrillation). As we previously described, morbidity was not related to the type of operation. As pneumonia is the most common complication in our study population, we would like to suggest to obtain preoperative sputum culture the day before operation these subset of patients due to possible colonization of microorganisms during oncological treatment process or preoperatively defined abscess condition.

Follow-up outcomes of several studies are shown in Table 2. Overall and relapse-free survivals were 23–46 and 5.9–43.6 months, respectively. Similar results have been reported in the study by Bauman et al. [5], wherein the survivals were 30 and 5 months, respectively. They concluded that early SLR in patients with abnormal fludeoxyglucose positron emission tomography scans resulted in higher survival rates than in patients with obvious relapse as seen using CT. Yang et al. [7] reported a median overall survival of 32.5 months. Patients with complete resection lived significantly longer compared with those with incomplete resection (60 vs. 20 months). Contrary to these encouraging results, Kuzmik et al. [6] reported a worse overall survival of 9 months.

We demonstrated that the mean overall survival was 19 ± 13 months, slightly lower than that reported in previous reports, and mean relapse-free survival was 14 ± 12 months, similar to that reported in previous studies. However, based on GI only, in which studies include only these patients, we had better results in agreement with the literature (22 and 16 months, respectively). There was no correlation with the survival rates and T- and N-stages of the tumor.

## Conclusion

Clinical experience with SLR remains limited, but the reports suggest that SLR is a worthwhile treatment with acceptable morbidity and low mortality rates. Identification of appropriate candidates for SLR has not been clarified and is challenging. We demonstrated that SLR is technically feasible when indicated and can be performed with acceptable mortality, morbidity, and long-term outcomes, even when anatomical resections greater than a lobectomy or extended resections are required. Patients with lower hematocrit and hemoglobin levels may suffer complications.

Based on our results, we suggest that surgery should be performed in patients with progression of the persistent primary tumor after definitive chemo- and/or radiotherapy. Despite the disappointing survival, other indications may be considered for SLR surgery based on the specific evaluation of each patient.

## Abbreviations

CT: computed tomography
DLCO: diffusion capacity of the lung for carbon monoxide
FEV1: forced expiratory volume in one second
GI: group 1
GII: group 2
GIII: group 3
SD: standart deviation
SLR: salvage lung surgery
VO2max: maximum volume of oxygen
